# Supplementary figures and images for: Developmental fine-tuning of medial superior olive neurons mitigates their predisposition to contralateral sound sources
Source: PLoS Biol. 2024 Apr 29;22(4):e3002586. doi: 10.1371/journal.pbio.3002586 (PMC11081505; doi:10.1371/journal.pbio.3002586)

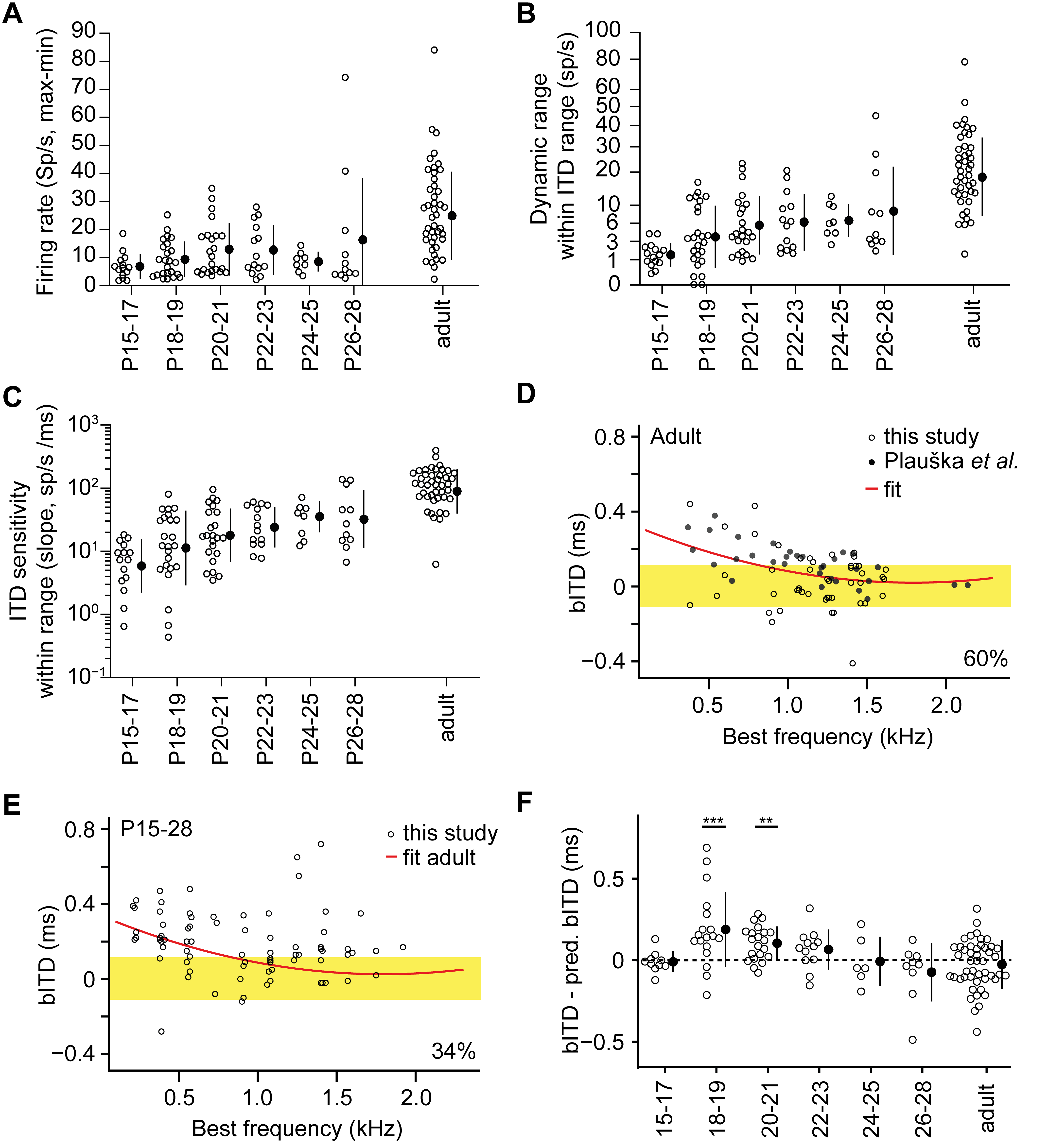

Supplement: S1 Fig — (A) Dynamic range of MSO firing at different ages. (B) Dynamic range within the adult ecological range of ITDs. (C) ITD sensitivity within the adult ecological range, quantified as the maximal slope of the ITD rate-curve (examples shown in Fig 2B). (D) bITD against the best frequency of adult MSO neurons. Data from reference [19] are reproduced here. Data were fitted by a quadratic function, giving bITD = β2 bFreq2 – β1 bFreq + β0, β2: 0.098 ms (kHz)−2, β1: 0.35 ms/kHz, and β0: 0.33 ms. Yellow box indicates adult ecological range. The number in the right bottom corner indicates the percentage of data points within the adult ecological range. (E) As D, but for juvenile MSO neurons. The fitted quadratic function in D is shown again for comparison. Yellow box indicates adult ecological range. The number in the right bottom corner again indicates the percentage of data points within the adult ecological range. (F) The difference between bITD and the predicted bITD against the age of the gerbil. The predicted bITD was calculated from the best frequency of the neuron and the quadratic equation in (D). F6,117 = 6.0, p = 1.5 10−5. Student’s T test to test if means differ from 0: *** P18-19, t17 = 5.1, p = 9.6 10−5, * P20-21, t20 = 3.2, p = 0.0041. The data underlying this figure is available at https://doi.org/10.5281/zenodo.10729468. (TIF) [file pbio.3002586.s001.tif]

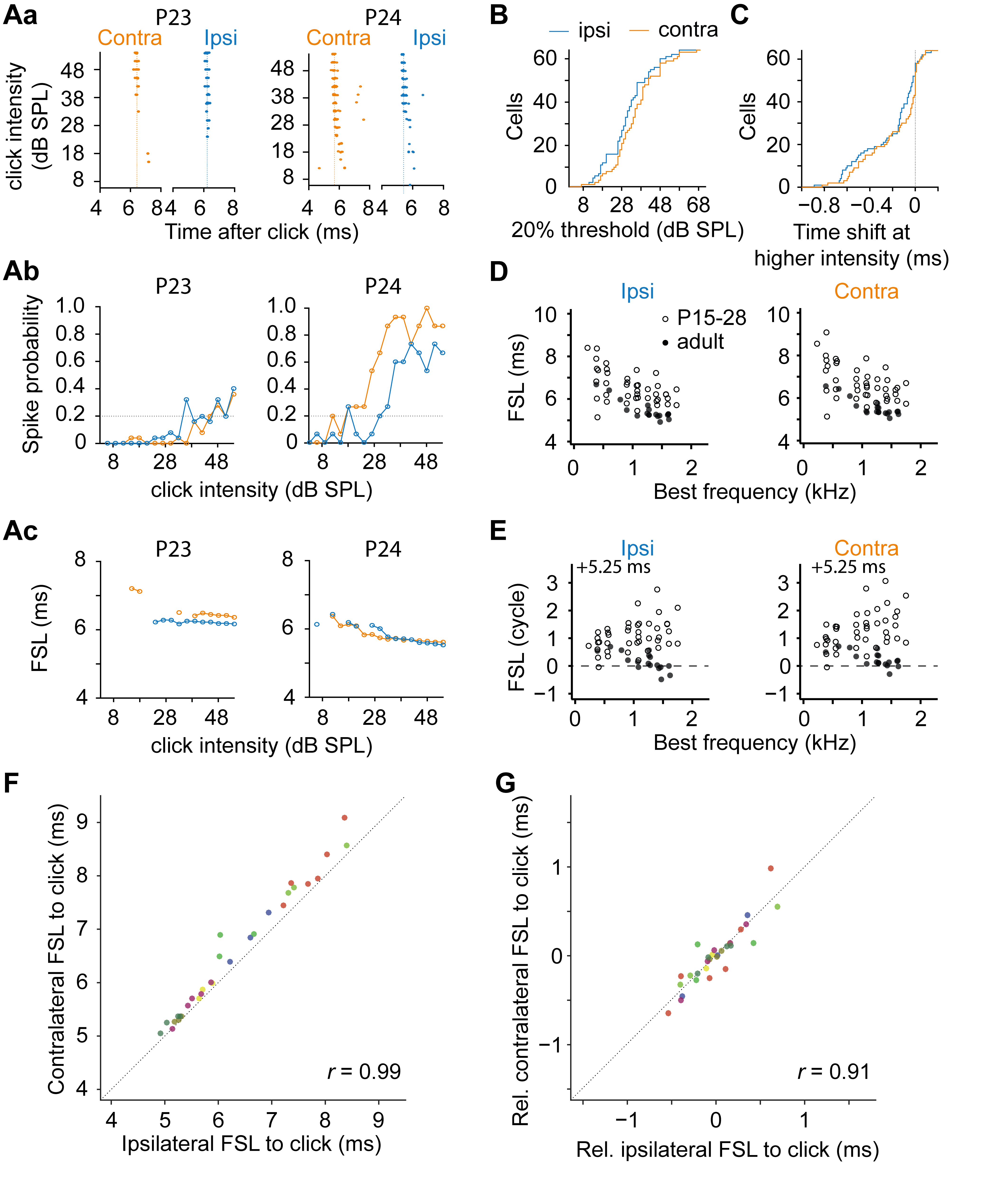

Supplement: S2 Fig — (Aa) Spike raster plot of a P23 MSO neuron and of a P24 MSO neuron for contralateral (orange) and ipsilateral (blue) clicks ordered along the vertical axis by click intensity. Median FSL is indicated as a dotted line. (Ab) Spike probability against click intensity of the same neurons as in (Aa). (Ac) Median FSL against click intensity of the same neurons as in (Aa). (B) Cumulative distribution of 20% spike probability threshold for juvenile MSO neurons. (C) Cumulative distribution of the time shift of the median FSL compared to the median FSL of the lowest click intensity that elicited ≥3 eAPs. (D) Ipsilateral and contralateral first-spike latency (FSL) against best frequency of MSO neurons. (E) Ipsilateral and contralateral first-spike latency expressed in phase against best frequency of MSO neurons. Phase of 0 corresponds to +5.25 ms. (F) Contralateral against ipsilateral FSL to click of MSO neurons from gerbils in which we recorded ≥3 neurons (n = 23 cells from 6 juvenile animals, 7 cells from 2 adults). Color corresponds to the unique animal. (G) Contralateral against ipsilateral relative FSL. The grand average of either contralateral or ipsilateral FSL of the animal is subtracted from the absolute contralateral or ipsilateral FSL, respectively. Individual animals are grouped by color as in F. Pearson’s correlation coefficients (r) are shown in the graph. Dotted lines indicate identity lines. The data underlying this figure is available at https://doi.org/10.5281/zenodo.10729468. (TIF) [file pbio.3002586.s002.tif]

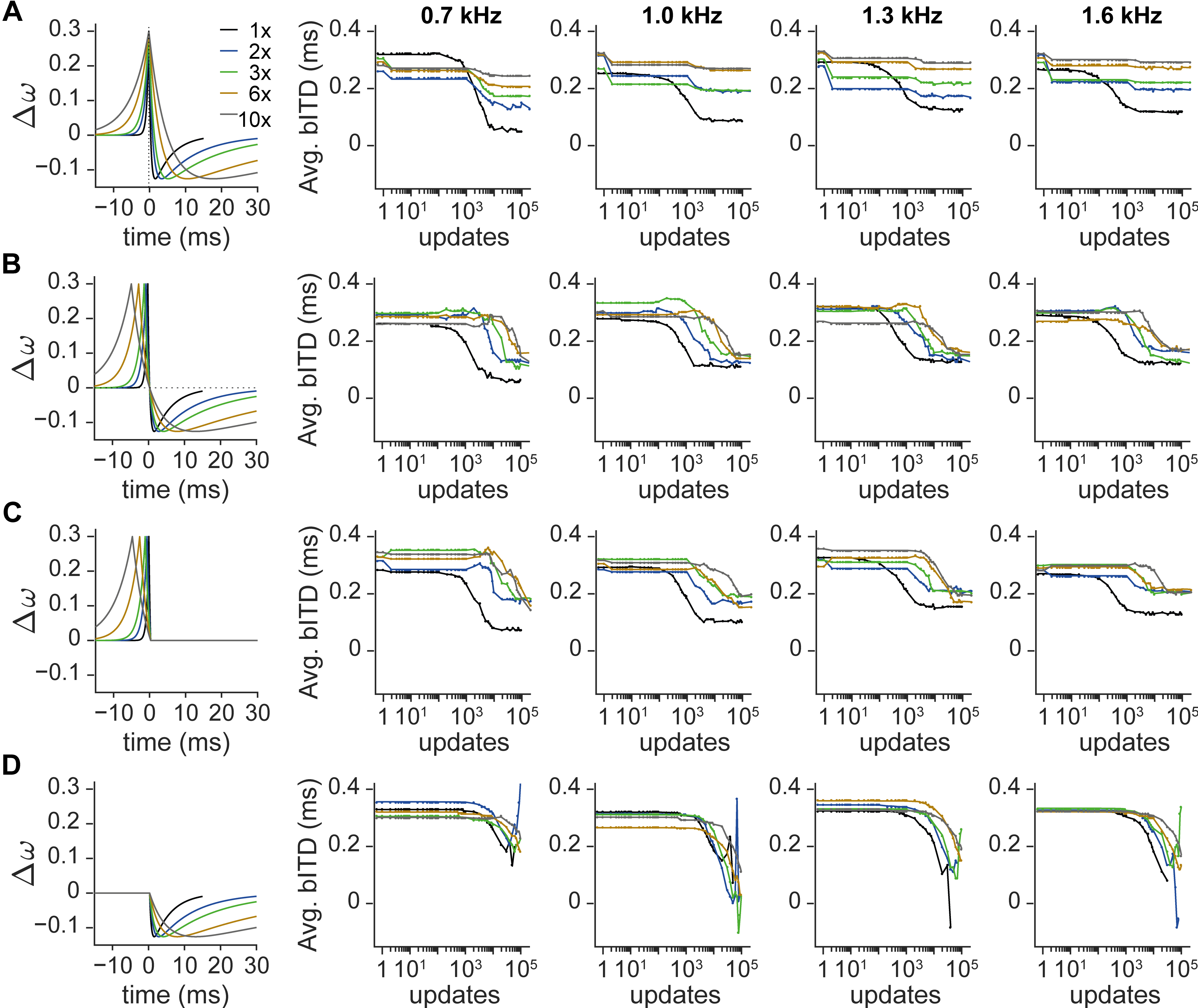

Supplement: S3 Fig — (A–D) Left column shows the broadened STDP rules and the right 4 columns show the average bITD against the number of updates of 400 neurons with a frequency tuning indicated on the top. (A) The broadened STDP rules were aligned on the peak of potentiation. (B) The broadened STDP rules were aligned on Δω = 0. (C) The broadened STDP rules aligned on Δω = 0 without the depression phase. (D) The broadened STDP rules aligned on Δω = 0 without the potentiation phase. In all conditions (A–D), a homeostatic mechanism kept the total synaptic weight constant. The code to generate this figure is available at https://doi.org/10.5281/zenodo.10729468. (TIF) [file pbio.3002586.s003.tif]

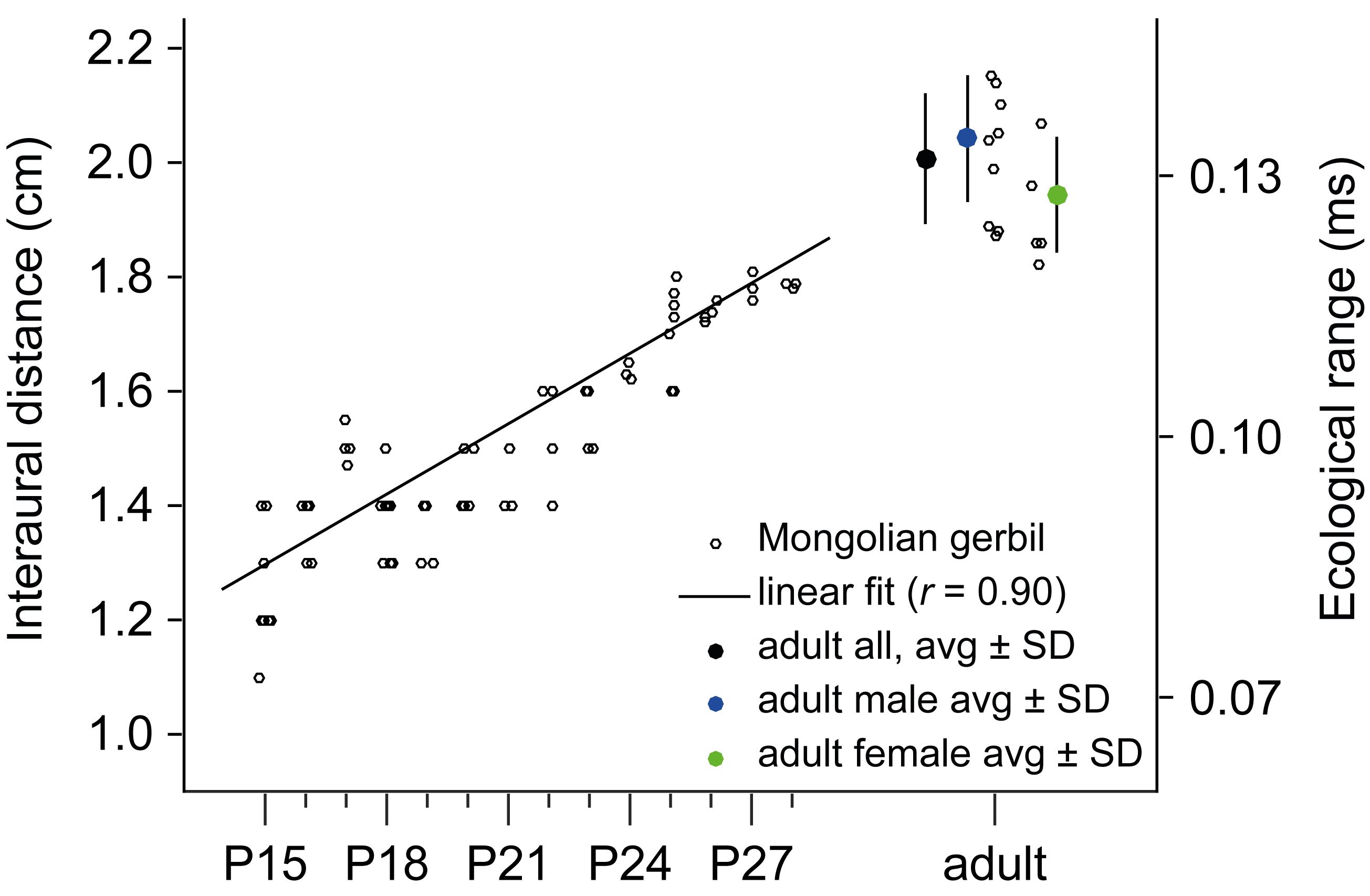

Supplement: S4 Fig — Developmental change in interaural distance (left axis). For the adult gerbil, the grand average (black), the male average (blue), and the female average (green) of the interaural distances are also shown. The ecological range (right axis) was calculated as the adult ecological range multiplied by the interaural distance normalized to the grand average of the interaural distances of the adult gerbils. The juvenile data was fitted by a linear function. The data underlying this figure is available at https://doi.org/10.5281/zenodo.10729468. (TIF) [file pbio.3002586.s004.tif]
