# Supplementary material for: Developmental fine-tuning of medial superior olive neurons mitigates their predisposition to contralateral sound sources
Source: PLoS Biol. 2024 Apr 29;22(4):e3002586. doi: 10.1371/journal.pbio.3002586 (PMC11081505; doi:10.1371/journal.pbio.3002586)
Supplement: Attachment — Submitted filename: Rebuttal Sierksma 2024.docx [file pbio.3002586.s006.docx]

REVIEWS:

We thank the three reviewers for their helpful suggestions to strengthen our paper and their concerns, which we addressed in our revised manuscript and in the point-by-point rebuttal below. We hope that the current version of our paper is acceptable for publication in PLOS Biology.

Reviewer #1, Jose L Pena (note, reviewer 1 has signed this review): Very good novel description and analysis of response properties of MSO neurons in juvenile Gerbils and developmental changes of these response properties determining response patterns in this brain region in adult animals.

Potentially significant model of spike timing dependent plasticity, mediating developmental changes of tuning properties and relationship between ITD and frequency tuning of MSO neurons in normal adult gerbils.

Strong results indicating investigated dendritic latencies versus upstream delay lines underlying mechanisms of ITD detection and indicating stronger role of delay lines in ITD tuning of MSO neurons.

Good citation of previous publications.

Overall, a very good manuscript and a few issues and suggestions for clarifying this study:

We thank Professor Jose Pena for his kind remarks and his suggestions for improvement.

Figures 2 and 3 indicate 'putative' ecological range of ITD in juveniles and adult gerbils, the Methods section indicates that ITD's ecological range was considered shorter in juvenile than adult gerbils based on changes in head diameter, and reported results of proposed STDP model show that when neurons were trained with different ecological ITD ranges, their best ITD changes shifted. However, previous publications supporting these assumed differences in ITD ecological range along development do not appear clearly cited and whether the change in ecological range has a potential effect on developmental changes in response properties and proposed STDP model is not clearly addressed in the manuscript.

We added a citation to the paper by Carr and Boudreau (1996), who measured the increase in head width during owl development and related this increase to the development of the sound localization circuit.

To our knowledge, no study has addressed the change in the ecological range during development of Mongolian gerbils. We therefore measured the interaural distance of the pups to allow an estimate of the ecological range at the different ages from the adult gerbil (Rebuttal Figure 1). We added this graph to the Methods-section as Figure S4.

**Rebuttal Figure 1 (= new Figure S4). Ecological range of ITDs of juvenile gerbils.** *The interaural distance against the age of the animal. For the adult gerbil the grand average (black), the male average (blue) and the female average (green) are shown. The ecological range is calculated as the adult ecological range, reported by Maki (reference [1] in our revised manuscript), divided by the grand average of the adult gerbils times the interaural distance of the animal. The juvenile data was fitted by a linear function.*

We did not speculate on the impact of a smaller ecological range on the tuning by STDP, but we have included a sentence in the Discussion to emphasize that the ecological range of developing gerbils allows even less compensation by acoustic ITDs (line 406).

Lines 112-114: Description of Figure 1E should specifically mention whether this is the spike-raster plot of same neuron as in 1D.

Done

Lines 179-180: Reading description of Figure 3B might become easier if colors representing value pairs for SFL of both ipsilateral and contralateral sounds are specified.

We added this information to the Legend of Figure 3.

Lines 220-223: Figure 4A is never called and maybe useful to call it in this part of the text, when mentioning the STDP modeling.

Figure 4A is now called at line 255.

Lines 271-273: a relationship between preferred frequency and ITDs was reported in the midbrain, not ITD detection neurons, of owls. This relationship trend is consistent with the observation that best ITDs become larger for lower frequencies. However in owls, best ITDs are not strongly outside the ecological range. Citing this report supports commonalities of ITD coding properties across species, however it may be useful to specify in the sentence on line 273 that "A relation between frequency and ITD tuning was also found in the owls' midbrain (47)."

We have adjusted the sentence as the reviewer suggested (line 388)

Line 639: Correct apparent typo in legend of supplementary figure S1, where this line should be calling panel F, not E.

Done.

Reviewer #2, Michael T. Roberts (note, reviewer 2 has signed this review): This study addresses the important question of how the interaural time difference (ITD) tuning of MSO neurons arises in the weeks following hearing onset. Using in vivo recordings in gerbils, the authors show that the best ITDs (bITDs) of MSO neurons move from well outside the ecological range to within the ecological range over the first two weeks following hearing onset. This shift correlates with a developmental decrease in the difference between the response latencies to ipsilateral and contralateral monaural stimuli within individual MSO neurons, suggesting that the travel times for ipsilateral and contralateral signals to reach MSO neurons are a main driver of ITD tuning. These results further suggest that a developmental mechanism exists to select for similar response latencies between ipsilateral and contralateral inputs. Using a computational model, the authors propose that spike-timing dependent plasticity (STDP) could provide such a mechanism. The model has the additional appeal of providing a potential explanation for why low frequency MSO neurons in mature animals are more likely to have bITDs outside the ecological range than high frequency MSO neurons. Overall, the experiments are elegant and compelling and convincingly support the main conclusions of the study. Some concerns about the authors' interpretation of EPSP to AP latencies, a question of whether an STDP mechanisms is biophysically realistic in MSO neurons, and the need for more clarity about the analyses conducted detract from the study. However, if the authors can satisfactorily address these concerns, the results would represent a significant advance on a problem of central importance to sensory science.

We thank Dr. Michael Roberts for this thorough review and appreciate his concerns with the paper which we hope to have addressed.

Major concerns

1. I found the arguments in the "EPSP-AP latencies contribute little to bITD tuning" section difficult to follow, and I believe much of my confusion came from trying to understand how EPSP-AP latencies relate to "the delay that occurs with dendritic propagation" (lines 199-200) or "dendritic latencies" (lines 208, 212). Since the experiments used a single recording electrode, I don't see how the authors can make any direct claims about dendritic latencies based on EPSP-AP latencies. I can see where comparing the slopes of the EPSPs might be informative, as the authors' lab did in their 2013 study (Figure 7 of van der Heijden et al.). This, combined with the EPSP-AP latency measurements might be useful, but on their own, the latency measurements are subject to other interpretations that undercut the authors' dendritic latency argument. Perhaps I am just missing the point here, but if that is the case, I encourage the authors to lay out their arguments more directly and clearly.

*Relation between dendritic and EPSP-AP latencies.*

We agree with the reviewer that in our experiments the dendritic latencies were not measured directly. However, in dendritic patch recordings or simulations (*e.g.* references [7, 42, 46] in our revised manuscript) the onset of the somatic EPSP is virtually simultaneous with the dendritic EPSP, while the peak/half-max of the somatic EPSP is much more delayed compared to the dendritic EPSP. We reproduced this (virtually) simultaneous onset with an adaptation of the model of Zhou et al. (reference [47] in our revised manuscript, Rebuttal figure 2). This model MSO neuron had two 200 micron-long dendrites: one thin dendrite with a diameter of 2 μm and one thick dendrite with a diameter of 5 μm, corresponding to measurements of MSO dendrites (reference [5] in our revised manuscript). On each dendrite, a synapse was placed at 100 μm from the soma, and the figure shows the corresponding EPSP recorded both at the synaptic location and at the soma. The eEPSP onset differed by 84 and 56 μs for the thin and the thick dendrite, respectively, compared to the onset at the synaptic location. This delay thus equals 0.56-0.84 μs/μm. For synapses closer to the soma, the dendritic latency will be even less. We therefore believe that the error in the measured eEPSP-AP latency is estimated to be within 80 μs. Similarly, the error in the difference in the ipsi- and contralateral eEPSP-AP latencies will be in the same range. We therefore assume that the onset time of the somatic EPSP is approximately the onset of the dendritic EPSP. With the EPSP-AP latency we then measure the dendritic propagation together with the spike triggering latency and latency for AP backpropagation.

At lines 214-244 we now lay out our assumptions more clearly, making it easier to evaluate their correctness by the reader.

**Rebuttal figure 2. Simulation of the dendritic latency measured as the difference in EPSP onset at the dendrite and in a juxtacellular recording.** *The simulated somatic recording was transformed to a juxtacellular recording using a resistive scaling factor of 0.127 mV/mV and a capacitive scaling factor of 5.6 μV/ (V/s) (reference [10] in our revised manuscript). The top row shows the simulated traces and the bottom row the same traces normalized to their peak potential. For the left panels a synapse and a recording electrode were placed on a 2-μm thick dendrite at a distance of 100 μm from the soma. For the right panels the synapse and the recording electrode were placed at the same distance on a 5 μm-dendrite.*

*Our arguments for the contribution of the EPSP-AP latencies to the tuning of bITDs*

Both the development of interaural difference in FSLs and the binaural difference in eEPSP-AP latency were correlated with the bITD (*r* = 0.71 and 0.73, respectively). That these factors correlate well with bITDs does not indicate their relative contribution to the bITD. The slope factor of the linear regression gives a better indication of this contribution. The slope factors were 0.69 ± 0.05 ms bITD/ms ΔFSL and 3.3 ms bITD/ms ΔeEPSP-AP. As its slope factor is <1, ΔFSL ‘overshoots’ the bITD (0.100 ms ΔFSL = 0.069 ms bITD), suggesting that some other processes may compensate for this overshoot. As the slope factor of ΔeEPSP-AP is >0, ΔeEPSP-AP is not compensating for the overshoot. The positive slope factor indicates that it explains a part of the bITD (~100%/3.3 = 30%), so 70% of the bITD is related to other latency differences. Whereas on the one hand we believe this analysis shows that EPSP-AP latencies make only a minor contribution to the bITD tuning, we also acknowledge that the sub-heading of this paragraph may cause confusion and may give the impression that the eEPSP-AP is not important, so we adjusted it to ‘Upstream latencies dictate bITD tuning of juvenile MSO neurons’.

2. The STDP model appears to have a lot of explanatory power and, on its surface, provides a compelling solution to the problem of how individual MSO neurons end up being driven by ipsi and contra afferents with similar FSLs. However, I am concerned that there is no evidence that STDP takes place in MSO neurons, and it is not clear if biophysical mechanisms exist that could support the presumably very brief timing rules needed for STDP driven by binaural stimuli with ITDs <0.13 ms. Most STDP timing rules extend over many milliseconds. Even in the DCN, where the timing rules are relatively brief, the window for LTP is still >10 ms (Tzounopoulos et al., 2004). The authors should explicitly acknowledge this caveat, clearly state or show the time window needed for the learning rules in their STDP model (this might be buried in the parameters, but it should be easy for the reader to pull out), and make it clear to the reader that the mechanisms to support STDP with such a brief timing rule are unknown. At the same time, I commend the authors for clearly explaining in the discussion that there are alternatives to STDP that could explain the refinement of binaural latencies.

We agree with the reviewer that we have not provided strong evidence for STDP in MSO cells nor a biophysical mechanism that supports very brief timing rules.

It was not our main goal to develop a new STDP model. We instead started with the STDP rule published by Gerstner *et al.* (reference [28] in our revised manuscript). This STDP rule features a sharp transition of ~2 ms from max. LTP to max. LTD. While this transition is briefer than reported in physiological experiments, sharp STDP curves have been reported:
 -Bi and Poo (1998) report a narrow transition range from maximal depression to maximal potentiation of ~5 ms around 0 ms pairing, which they describe mathematically as an instantaneous switch from LTP to LTD, RT.
 -Debanne et al. (1998), +15 ms potentiation, 0 ms depression, 32 °C.

- Feldman (2000), +3 ms potentiation, -10 ms depression, RT.
 -Sjöström et al. (2001), +10 ms potentiation, -10 ms depression, 32 °C.

-Tzounopoulos et al. (2004), +10 ms potentiation, -10 ms depression, RT (reference [49] in our revised manuscript).
 -Froemke et al. (2005), a transition in less than 10 ms, RT.

-D'amour and Froemke (2015) within -10 and +10 ms from depression to potentiation, 33 °C.

Some of these experiments were performed at RT, and we expect that these windows would be even narrower at physiological temperatures. We extended our simulations to investigate whether a more physiological time window could still reproduce the shift in bITDs. With broader STDP rules (2, 3, 6 or 10 times) and an increased number of updates (Rebuttal figure 3A, new Figure S3) we first found that these broadened STDP rules were ineffective to create the same shift in the bITDs, but we hypothesized that this outcome may have been related to the newly-introduced potentiation of synapses that were active after the AP, unlike the original STDP rule, where synapses active after the AP would become depressed. By realigning the broadened STDP rules to the transition from potentiation to depression, the shift in bITDs was retrieved (Rebuttal figure 3B). Although the simulations needed more updates the broader the STDP rule became, the shift was generated nonetheless. We therefore conclude that, in principle, broader STDP rules can still shift the bITDs toward the ecological range using absolute ITDs <0.13 ms.

**Rebuttal figure 3 (= new Figure S3). The shift in bITDs in models with different spike timing-dependent plasticity rules and homeostasis.** *(A-D) Left, the actual STDP rules used in the simulations. Right, the average shift in bITDs of 400 neurons tuned to 0.7, 1.0, 1.3 and 1.6 kHz against the number of updates using the STDP rules shown in the left panel. (A) The broadened STDP rules were aligned on the peak of potentiation. (B) The broadened STDP rules were aligned on Δω = 0. (C) The broadened STDP rules aligned on Δω = 0 without the depression phase. (D) The broadened STDP rules aligned on Δω = 0 without the potentiation phase.*

As our model includes a homeostatic mechanism that maintains a constant total synaptic weight, we also tested whether the STDP rule could be partially replaced by a rule that included the homeostasis and either only the potentiation phase of STDP or only the depression phase of STDP (panel C and D, respectively). The shift in bITDs was still present after more updates to attain the same shift, but the outcome of the simulation was unstable without the potentiation phase as the model neurons lost all but one synapse in many cases. From these simulations we conclude that broadened, more physiological STDP rules also generate a bITD shift as long as well-timed, co-active synapses are potentiated and asynchronous synapses are depressed.

We added to the text that evidence for STDP in MSO neurons is currently lacking (line 354) plus the new simulation results (lines 292-305), and we now discuss STDP and point to a possible starting point for the underlying mechanism of STDP in MSO cells (lines 366-385). The reviewer requested that we would show the STDP rules, which we now provide in Figure S3.

Minor concerns

1. Results, line 81. Since the Methods section does not come until the end of the paper, it would help the reader here to clearly specify the age range meant by "juvenile" and to indicate that recordings were made using the juxtacellular approach.

We adjusted the first paragraph of Results to make it clear what we mean by juvenile gerbils and indicate what type of recordings we have made.

2. Figure 2C and 3C and Results, line 399. The yellow band expands with age because the gerbil head size increases with age, but how did the authors decide on the parameters to use for this? Did the authors measure head sizes themselves or did they base these measurements on a previously published report? In addition, no citation is given on line 399 where the authors describe the change in head diameter.

In our submitted manuscript we assumed that the ecological range was smaller than in the adult, and the reported values were our best guess. In our revised manuscript we measured the developmental increase in the interaural distance of the pups. For the convenience of the reviewer, we show below again rebuttal figure 1.

**Rebuttal Figure 1 (= Figure S4). Ecological range of ITDs of juvenile gerbils.** *The interaural distance against the age of the animal. For the adult gerbil the grand average (black), the male average (blue) and the female average (green) are shown. The ecological range is calculated as the adult ecological range, reported by Maki (reference [1] in our revised manuscript), divided by the grand average of the adult gerbils times the interaural distance of the animal. The juvenile data was fitted by a linear function.*

3. Results, line 165: I found the phrase "which relates to a frequency-dependent latency" difficult to follow until reviewing the referenced figure (S2D-E). It would be more reader friendly to directly state what the relationship is.

We added that latencies were smaller for neurons tuned to higher frequencies, and we have added the empirical slope value of the linear regression (lines 179-181).

4. Results, line 167: It is not clear how "within-animal SD" was calculated. Is this a mean of standard deviations across animals?

The reviewer is correct that the SD was calculated per animal and we describe the average SD and the SD for the SD. We adjusted the text to make this clear.

5. Results, lines 197-198. The reader needs more info here to understand where the calculated SD of 0.4 ms comes from (i.e., what are the SDs or variances of the ipsi and contra FSLs; I did not see this clearly stated anywhere). Please also directly state the SD of the delta FSL that was observed.

In lines 214-219 we write the SDs of the ipsi and the contra FSLs, which were both 0.3 ± 0.2 ms. For each animal we can then calculate an expected SD for ΔFSL as √(SD_ipsi_^2^ + SD_contra_^2^), which gives 0.43 ± 0.25 ms. This expected SD is higher than the measured SD for ΔFSL, which is 0.12 ± 0.10 ms.

6. Results, line 201 and Methods, line 426. Please standardize how you refer to the seal resistance of the juxtacellular recording. It is described as "input resistance" in the results and "bridge resistance" in the Methods. To me, "seal resistance" would be the preferred term since input resistance is generally synonymous with membrane resistance and bridge resistance is generally an indicator of electrode/series resistance.

We now use “seal resistance” both in the Results and in the Methods.

7. Figure 4 legend, lines 253-254. The authors state, "Neurons with lower best frequencies need more updates to adjust their bITDs to the training ITDs." Did the authors use the same stimulus duration for low and high frequency stimuli? This would be reasonable, but it also means that high frequency stimuli generate more opportunities for EPSP-AP pairing. Please address this point to make it clear to the reader.

As a stimulus in the model we uses an idealized click stimulus, which avoids phase-locking, entrainment, short-term plasticity, intrinsic plasticity and inhibition, which are likely to play a role with ongoing sounds. Briefly, the impulse activates the inputs from one ear at once and their postsynaptic EPSP is delayed by their specific latencies.

The reviewer’s suggestion that neurons sensitive to high-frequency tones may have increased opportunity for EPSP-AP pairing with ongoing sounds is an interesting idea which is not incorporated in the model. This will depend on entrainment, which may be higher again at lower frequencies. We similarly suggested that cycle-skipping increases the chance of EPSPs overlapping, which is currently not included in our model. As the goal of the model is to address whether STDP can adjust ITDs to the ITDs used for the training despite an initial bias to contralateral-leading ITDs, we prefer to keep the model as simple as possible by using an idealized click.

We added to the Results section that we used an idealized click stimulus to activate the inputs and that their timing depended on their specific latency, which included an ITD.

8. Methods, line 425. Typo - "a" should presumably be "(".

Corrected

9. Figure S1 legend, line 633. Typo - even though it starts a sentence, I believe "BITD" should be "bITD".

Corrected

10. Figure S1 legend, line 639. Typo - "(E)" should be "(F)".

Corrected

Reviewer #3: In this study, Sierksma & Borst quantified the distribution of the ITD that elicits peak response rates (bITD) from neurons of the MSO in the gerbil at different developmental stages. The MSO is the primary nucleus for the neural computation of ITD, i.e., the time difference in the arrival of a sound at the two ears, which is the primary cue for sound localization of low frequency sounds. It is by now well established that the bITD in mature mammals, including gerbils, depends on the best frequency tuning of the cell: the lower the best frequency, the larger (more contralateral) the bITD of the neurons. Importantly, for best frequencies below approx. 1kHz, bITDs typically lie outside the physiological range (the max. ITD that the inter-ear distance can generate). In this study, the authors asked how / to what extent this contralateral bITD preference develops during the maturation of the animal. They report that a strong contralateral bias of bITDs can be found at early stages after ear-opening (P15 to P23), which apparently vanishes in more mature age groups. Based on the analysis of monaural latency calculations, they show that bITDs are consistent with predictions of differences in the conduction delays between ipsi and contralateral inputs. The authors then ask about potential mechanisms for this apparent alteration of bITD distributions during maturation. To this end, they provide data from a STDP model, suggesting that the relative timing between pre-. and postsynaptic spiking can contribute to bITD tuning.

The question of ITD tuning of MSO neurons and the underlying mechanism have been a topic of investigation for decades and is still not fully understood. Hence, studies regarding its development are of general interest. However, in my evaluation, the study falls short of providing conclusive or novel insight due to a number of reasons:

We thank the reviewer for stating that our manuscript is potentially of general interest, and we hope that we have addressed his/her concerns in our revised manuscript.

Major concerns:

1) Most critically, besides a lack of consideration of existing literature (see below), the significance of the frequency-dependency of bITD and related coding capacity of the neurons is mostly disregarded and the number of neurons in the most relevant frequency range (below 1kHz) is too low to allow making conclusions. In fact, the only information about frequency tuning is provided in the Suppl. Figures: Given this limited information that is provided, I conclude that the effect of large bITDs shifting towards the physiological range with age is dominated by neurons tuned to higher frequencies (above 1 kHz. Suppl. Fig. 1D,E). I.e., for cells below 1 kHz the internal delay seems to be similar to what is expected in the adult, and a maturation is observable only in higher frequency cells. In fact it seems that in the current data set only one neuron below 1 kHz (out of seven) in adult gerbils exhibited large contralateral leading bITD, which contradicts the strong contra bias reported in existing data from the gerbil MSO, including data from the authors' lab. Thus, by not providing bITDs separately for lower and higher frequencies, the data shown is Fig. 2 is actually rather misleading.

*MSO neurons tuned to >1 kHz are relevant for sound localization.*

In major concern 1 and 2 the reviewer indicates that the ‘most relevant’ frequency range is below 1 kHz, indicating that neurons tuned to above 1 kHz are less relevant for sound localization. MSO neurons are sensitive to ITDs because of phase-locked firing of spherical bushy cells (SBCs). In gerbils, phase-locking of SBC occurs for tones up to 3 kHz (Dehmel et al. 2010; Kuenzel et al. 2011). MSO neurons can therefore in principle compute the ITD for tones up to 3 kHz. The computation of ITDs above 1 kHz does have clear behavioral relevance. The work of Tolnai, Beutelmann & Klump (references [3, 4] in our revised manuscript) indicated that gerbils have the highest sensitivity to ITDs at 1 kHz and could detect ITDs well in 2 kHz tones, in agreement with the electrophysiological results. Furthermore, the minimal resolvable angle of Mongolian gerbils was found with tones at 2 kHz (reference [2] in our revised manuscript). MSO neurons that detect ITDs for tones of 1-2 kHz are therefore expected to contribute to the ability to localize sounds. We added a sentence to the introduction to indicate that the Mongolian gerbil detects ITDs of sound frequencies up to 2 kHz.

*Juvenile and adult low-frequency neurons have a significantly different bITD.*

We conducted additional experiments to increase the number of low-frequency neurons and to test if they are different from the high-frequency neurons. We recorded from twenty additional cells, of which six neurons were tuned to frequencies <1 kHz. The adult data set then included fourteen cells tuned to <1 kHz out of 49 cells of which four cells had a bITD >0.26 ms. These fourteen cells had a bITD of 0.09 ± 0.21 ms (min: -0.19 ms, max: 0.44 ms), which was significantly different from the juvenile cells (0.24 ± 0.13 ms, Welch’s *t*_17_ = 2.39, *p* = 0.03). The frequency tuning of these neurons is presented in the main text and the supplementary figure S2 is updated with the new data. Figure S2D also shows bITD against frequency and includes a comparison of our current study with our previous work (Plauska et al. 2017, reference [19] in our revised manuscript).

As the reviewer prefers the bITDs of the groups to be reported separately, we added in the text the bITDs for MSO neurons tuned to either <1.0 kHz or ≥1.0 kHz (lines 147-155) as well as the maturation effect for both groups. As both groups show a shift in bITDs in the same direction and the criterion of 1 kHz to separate these two groups of MSO neurons is rather *ad hoc* and arbitrary, the presentation of the pooled data in the figures 2 and S2 in our opinion is the best way to present the data to the reader and, with the added text, allows the reader to judge the validity of our claims. As we agree with the reviewer that the shift in bITD could be less for neurons tuned to very low frequencies, we state in the Results that this may be the case (lines 154-155).

In any case, these observations lead to important follow-up questions that were however not addressed: large bITDs in juvenile gerbils contradict an earlier report (Seidl & Grothe 2005) of bITDs close to midline in the juvenile gerbil (in DNLL and IC). Yet this discrepancy is not even mentioned, led alone discussed.

*The data of Seidl & Grothe are in line with our data, notwithstanding the opposite conclusion*

We did cite this paper at lines 272 and 291 in our submitted manuscript for the observation that also in the DNLL (Seidl and Grothe did not record from the inferior colliculus) “ITD tuning for low frequencies, especially for small mammals, lies outside the ecological range, whereas at high frequencies, for most cells it lies within”. We agree with the reviewer that we could have discussed the developmental findings in that paper as well, especially since, as noted by the reviewer, the authors draw conclusions that are opposite to ours. Seidl & Grothe (2005, reference [51] in our revised manuscript) recorded from 76 cells at the age of P15, of which 9 cells matched their selection criteria. These 9 cells had an IPD tuning of 0.082 ± 0.074 cycles. Interestingly, this does not significantly differ from what we recorded, as we have a bIPD for P15-17 cells of 0.067 ± 0.071 cycles (n = 10 cells), which gives a t-score of 0.26 and a p-value of 0.80 that there is no difference in bIPD between their work and ours. In our study the difference with the adult bITDs becomes most obvious at P18-21, whereas Seidl & Grothe only recorded from P15 and adults. As we made a comprehensive timeline of the development of ITD tuning, we find that our data are consistent with the data of Seidl & Grothe (2005), while uncovering the interesting tuning that occurs during the period following hearing onset. The Seidl & Grothe paper does not report the relationship between ITD and frequency tuning for DNLL neurons, but this is reported for the adult neurons in a later paper from the same group, which also included the data of Seidl and Grothe (Figure 9A of Siveke et al., 2006, reference [56] in our revised manuscript). It appears similar to the results we found for the adult MSO: above 0.6 kHz, the majority of these neurons (32 out of 58 cells) are tuned within the ecological range, while below 0.6 kHz, the majority lies outside this range (17 out of 20 cells). Despite the somewhat uncertain relationship between the tuning of MSO and DNLL neurons, we therefore do not see a clear discrepancy between the two for both the adult and the juvenile situation. We have added a summary of the above arguments to the Discussion (lines 318-339).

Additionally, the fact that frequency tuning is estimated based on vector strength in response to a complex stimulus (Zwuis stimuli) instead of pure tones complicates matters further and could contribute to the rather unusual distributions.

*Zwuis has clear advantages over pure tones*

We have addressed the remark about the unusualness of our distributions in our answer to the previous comment.

As we do not know the frequency tuning of an MSO neuron beforehand, the Zwuis has the advantage that often some of the primary Zwuis frequencies fall within the frequency tuning of the neuron and therefore activate the neuron. MSO neurons can show broad frequency tuning and ITD tuning (references [9-11, 14, 17, 20, 21, 53] in our revised manuscript), making the Zwuis stimulus well-suited for studies of the MSO. A pure tone may not drive MSO neurons significantly unless it activates multiple spherical bushy cells. One way to achieve activation of many SBCs with a pure tone is by moderate-intensity, low-frequency tones which activate many spherical bushy cells at their low-frequency tail of their receptive field (*e.g.* (Kuenzel et al. 2011)). While this is an effective stimulus to drive MSO neurons, off-frequency tone stimulation can introduce temporal delays in an unpredictable manner (Palmer and Shackleton 2009; Versteegh et al. 2011), which changes the ITD tuning curve of the MSO. Moderate-intensity tones may furthermore already lead to peak-splitting in the firing of bushy cells when stimulated in their low-frequency tail (reference [10] in our revised manuscript, and (Spirou et al. 1990; Joris et al. 1994; Wei et al. 2017)). These sudden shifts in the phase of bushy cell firing can radically change the ITD tuning of MSO cells. If during development the ITD tuning is entrained by natural sounds, then moderate-intensity low-frequency tones are not the most relevant stimulus to study MSO’s ITD tuning and may even obscure the relevant ITD tuning of the MSO neurons by introducing delays. Zwuis is also a more efficient stimulus, which is important since the juvenile recordings tended to be less stable than the adult ones. We therefore consider a low-intensity broadband stimulus, such as the Zwuis a defensible choice.

In the Method section we added a sentence to explain why we have chosen for a Zwuis stimulus.

2) The author repeatedly claim that neurons with bITDs inside the physiological range would represent "Jeffress-like peak-coding" cells, and only neurons with bITDs outside the phys. range are "slope coders". This claim is not validated and I'm not aware of any data supporting this claim, particularly not in a small-headed animals as the gerbil.

Harper, McAlpine & colleagues (2014) specifically report a uniform "slope code" for the gerbil. Accordingly, the shift towards or inside the phys. range is fully predicted by the frequency-dependency and associated slope position (e.g. compare reviews by Grothe et al., 2010; McAlpine 2005; Pecka & Encke 2020). If anything, one could discuss if or to what extent the high-F tuned neurons (which are responsible for the main effect of bITD change) provide important ITD information in general, but assuming a switch in the coding regime is unwarranted.

*Evidence for a developmental shift from slope coding to peak coding*

In our submitted manuscript we distinguish between ‘peak coding’ and ‘slope coding’ by defining ‘peak coders’ as neurons that are tuned to a specific sound source direction (line 52) and ‘slope coders’ as neurons that increase their firing as sounds are coming increasingly from the other side (line 60). In our experimental conditions where we can test for ITDs that are outside the ecological range, we observe then that some MSO neurons have a bITD within the ecological range (hence, peak-coders) and some outside the ecological range (hence: slope-coders).

For both slope-coding and peak-coding neurons the firing rate indicates how close the sound source is to their optimal sound source direction, and for both the sub-maximal firing rate within the ecological range may be ambiguous to which direction it points as the firing rate can correspond to a sound direction either more frontal or more caudal than the optimal sound source direction. The main coding difference is that the activity of slope coders all indicate how contralateral the sound direction is, while the activity of peak coders can indicate how nearby the direction of a sound source is to a particular direction. As we observe that many juvenile neurons have a bITD outside the ecological range (hence: slope coders), but in adult MSO neurons can either have bITD inside as well as outside the ecological range (hence: slope coders and peak coders), it suggests that some neurons have switched in how they encode sound direction during development. We added a sentence in the discussion to emphasize that the mechanism we envision entails this switch of some neurons from slope-coding to peak-coding (lines 316-317).

Harper and McAlpine (2004, reference [75] in our revised manuscript)) is a modelling paper, and does not contain actual measurements of ITD tuning in the gerbil MSO. Please also note that -in contrast to what the reviewer states- this paper only reports a uniform “slope code” for the gerbil for low (<1 kHz) frequencies, but for high frequencies a population of cells with a best IPD close to 0 cycles is additionally predicted (Fig. 2c of Harper and McAlpine, 2004). The optimal coding hypothesis of Harper and McAlpine (as well as Optimal coding of ITDs in McAlpine 2005 and The Neural Representation of ITD in Grothe et al. 2010) indicate that ITD-related information will be maximal if the firing of an MSO neuron discriminates between physiological ITDs. For any neuron with a broad tuning (relative to the ecological range), it is optimal to have a slope instead of its peak inside the ecological range. The optimal bITD would then be outside the ecological range. Optimally speaking, these reviews propose, all MSO neurons for gerbils should be slope-coding (dual-channel population coding) tuned to the contra or ipsilateral side. However, the publications in which neurons with a stark contralateral tuning were described, often find neurons that are tuned within the ecological range. According to the dual-channels model, these neurons should have a sharp tuning and respond to high frequencies, but this is not necessarily the case (for examples see Fig. S2D, reference [19] in our revised manuscript for gerbils, or for cats, see the examples in reference [15] in our revised manuscript, or for guinea pigs, see McAlpine et al. 1996 and reproduced in 2001 references [22, 23] in our revised manuscript). In our study we find that many MSO neurons are peak-coding, and that they nonetheless discriminate between ITDs within the ecological range (Fig. S1B and C).

The main topic of our paper is how the MSO neurons develop their ITD tuning. The reviews mentioned by the reviewer do not include a developmental mechanism that would arrive at the optimal coding scheme. We conclude that the bias to contralateral sound sources at the start of the tuning period may contribute to develop MSO tuning to something similar to the optimal coding scheme. We added a sentence to point out that the predisposition of MSO neurons creates the dual channels-model, and that the development fine-tuning of neural latencies may create a homogeneous representation of sound directions across MSO neurons at lines 389-399.

We also added, as mentioned in our answer to concern 1, to the Introduction that Mongolian gerbils can behaviorally detect ITDs in 2 kHz tones, which tacitly implies that the neurons that respond to these frequencies likely contribute to sound source localization, as requested by the reviewer.

As a side note, the optimal coding idea put forward by Harper & McAlpine (2004) disregards a number of conceptual problems (e.g. for broadband signals: how are different codes in a uniform population of neurons read out?) and is complicated by comparing data from different levels of processing (MSO vs downstream areas such as cortex).

We cited Harper & McAlpine (2004) because it highlights some of the advantages of slope coding, but we agree with the reviewer that this paper has its limitations as well.

3) Throughout the paper, the presence and influence of the inhibitory inputs to MSO is ignored. Inhibition has been implicated in an experience-dependent mechanism for bITD tuning during development (e.g Brand et al., 2002, Kapfer eta al., 2002), yet the authors don't discuss this influence nor do they include inhibition in their model. In fact, an inhibitory LTP mechanism for developmental refinement of ITD processing has recently been suggested (Winters & Golding 2018), but again is not considered by the authors. The apparent complete disregard of the anatomical and physiological data of MSO processing severely diminishes the relevance of the presented data.

Likewise, other developmental changes (width of frequency tuning / spectral integration etc.) are not considered either, further questioning the significance of the model data.

*Lack of evidence for a contribution of well-timed inhibition to ITD tuning*

The reviewer lists two influential articles by the Grothe group. Especially the Brand paper has dominated the debate (and many of the experiments) on ITD tuning in the MSO during the last 20 years. We did cite this paper and the related Pecka et al (2008) paper as a possible mechanism. Moreover, we have weighed in on this debate in each of our previous papers on MSO, admittedly squeezed into this by one of the reviewers in each case. At the request of the reviewer we have therefore added a discussion of the role of inhibition in ITD tuning of MSO neurons in our revised manuscript, including a citation of the relevant references (lines 330-339).

A summary of the argument against the role of well-timed inhibition in shifting bITD tuning to outside the ecological range, as proposed by Brand et al, 2002:

Most importantly, the main argument in favour of the well-timed inhibition was the shift in tuning towards 0 µs observed following strychnine, a blocker of glycine receptors. Franken et al (2015) showed that, at high concentrations, strychnine also blocks intrinsic conductances, most likely the cation channel *I*_h_. They concluded that “These experiments suggest that the best delay shift observed with strychnine iontophoresis^10,19^ is a result of nonspecific effects of strychnine at high concentrations and not to the removal of inhibition.” “Our data directly demonstrate that removing inhibition does not systematically shift the ITD function toward negative ITDs, contradicting the primary evidence for the inhibitory hypothesis^10,19^. Both our in vitro and in vivo data suggest that the previously reported effects of strychnine on best delay are probably mainly a result of nonspecific effects on intrinsic conductances”. Other arguments against an important role of well-timed inhibition on ITD tuning include the following. Well-timed inhibition was not observed during whole-cell in vivo recordings (van der Heijden et al, 2013; Franken et al. 2015). For a realistic time course of the IPSPs, the shift in ITD tuning will not work (Roberts et al, 2013). There is no evidence for a nonlinear interaction of the inputs of the two ears, as these inputs sum linearly (van der Heijden et al, 2013) and the binaural output of the MSO neuron can be well predicted by the monaural responses (Plauska et al, 2016; Mackenbach and Borst, 2023).

We therefore conclude that the changes in bITDs observed by Brand *et al* are unlikely the result of well-timed inhibition. Other important contributions of inhibition (e.g. sharpening the tuning curve, reducing the maximal firing rate) are likely to be present, but these contributions can neither be demonstrated nor disproven by our findings.

We have added to the Discussion a shortened version of the arguments against a major impact of well-timed synaptic inhibition on the bITD (lines 318-329).

We have also added a discussion of the Winter and Golding (2018) paper in our revised manuscript (lines 363-374).

Additionally, it is unclear to me how the frequency dependency of bITDs is achieved in the model. From the little information I found, less shifting during maturation for lower frequencies is realized by having less updates? That would bear no physiological relevance and again completely ignores existing literature.

*Low-frequency neurons need more updates to adjust their ITD tuning*

As we suggest that ITD tuning of a neuron is dominated by the upstream latencies and not by the intrinsic properties of the neuron, we designed a model in which every MSO neuron is the same (same STDP rule, same excitability, same number of inputs from each side, same unitary EPSP), except for the latencies of the synaptic inputs. Each MSO neuron is assigned a characteristic frequency (*f*_c_) which is only used to define the latencies of its synaptic inputs. The latency of a synaptic input includes a side-specific latency (ipsi: 5 ms, contra: 5.3 ms) plus two latencies that vary between inputs, namely a random latency (taken from a normal distribution centered at 0 ms and an SD of 0.3 ms) and a phase-dependent latency. This phase-dependent latency is defined by a random phase (a uniform distribution between 0.25 and 1) and the characteristic frequency of the synaptic input, which is randomly chosen from a uniform distribution between *f*_c_ − 0.5*f*_c_ and *f*_c_ + 0.5*f*_c_. This is the only contribution of frequency that is included in the model. The characteristic frequency of the neuron defines the latency distribution of the synaptic inputs of a neuron.

Each input generates an EPSP, and each EPSP sums linearly with the other EPSPs to obtain the membrane potential. The ITD changes the relative timing of the EPSPs generated by the synapses. The bITD is then defined as the ITD where the summed EPSP has the highest value. At initialization bITDs are scattered around +0.3 ms by how we defined the latencies (Figure 4E-F ‘hearing onset’). During the training phase the synaptic weights are updated based on the STDP rule and their relative timing to firing probability, which leads to a redistribution of synaptic weights among the synapses. The change in synaptic weights changes the bITD. As the STDP rule causes a larger change in weights for inputs that are activated together with the spike than for inputs that are more distant in time, neurons for which the synaptic inputs are active almost simultaneously and therefore closer to the ‘spike’ will rapidly change their synaptic weight. However, for low-frequency neurons the activity of the synaptic inputs are more distributed in time. As a result, for low-frequency neurons the synaptic weights make smaller changes with each update than those for high-frequency neurons. The smaller the changes in synaptic weights, the slower the synaptic weights develop and converge to an optimum. It is therefore not that some neurons have ‘less’ updates, (in fact the neurons undergo the same number of updates), but the synaptic weights of low-frequency neurons change with smaller steps per update due to the distribution of the timing of their inputs. If these neurons are given more updates, they converge nonetheless to their optimum (see Figure 4D and S3).

The physiological relevance is that the synaptic inputs of low-frequency neurons are less well-timed and therefore are less likely to show temporal overlap during brief sounds. As temporal overlap is necessary to get the MSO neuron to spike and to elicit STDP, and as STDP only has a strong effect on contemporaneously active synapses, less STDP is elicited in low-frequency neurons and therefore the tuning of inputs of low-frequency neurons to ITDs takes more time. A shortened version of these arguments is presented in the Discussion (lines 397-414)

While the frequency-dependency in our model is interesting, the main point we want to make is that the tuning of bITDs to the ecological range still occurs in spite of an initial bias in the contralateral latency, indicating that STDP is a viable mechanism for the developmental changes we described in this manuscript.

4) The quantifications of bITDs n Fig. 2 seem to based on very small sampling sizes: it is stated in the Methods that as little as four repetitions were required for the construction of rate-ITD functions. Such low repetitions hardly allow for reliably estimates of mean rates / bITDs.

To determine the bITD we used the Zwuis stimulus and systematically varied the ITD. In a random order we presented ITDs from -2 to +2 ms with a 0.1 ms interval, so we obtain 41 data points per repetition. We used four to ten repetitions, giving us 164 to 410 firing rates. These firing rates were then fitted by a power-transformed Gabor function. The examples we show of ITD tuning are representative, showing that the number of repetitions in our measurements was sufficiently large. Moreover, If the Gabor fit was not significant (*p* > 0.001), we excluded the neuron from our analyses on ITD.

**References specific to the rebuttal**

Bi GQ and Poo MM (1998). Synaptic modifications in cultured hippocampal neurons: dependence on spike timing, synaptic strength, and postsynaptic cell type. *J Neurosci* **18**: 10464-10472 <https://doi.org/10.1523/jneurosci.18-24-10464.1998>.

D'amour JA and Froemke RC (2015). Inhibitory and excitatory spike-timing-dependent plasticity in the auditory cortex. *Neuron* **86**: 514-528 <https://doi.org/10.1016/j.neuron.2015.03.014>.

Debanne D, Gähwiler BH and Thompson SM (1998). Long-term synaptic plasticity between pairs of individual CA3 pyramidal cells in rat hippocampal slice cultures. *The Journal of Physiology* **507**: 237-247 <https://doi.org/10.1111/j.1469-7793.1998.237bu.x>.

Dehmel S, Kopp-Scheinpflug C, Weick M, Dörrscheidt GJ and Rübsamen R (2010). Transmission of phase-coupling accuracy from the auditory nerve to spherical bushy cells in the Mongolian gerbil. *Hear Res* **268**: 234-249 <https://doi.org/10.1016/j.heares.2010.06.005>.

Feldman DE (2000). Timing-based LTP and LTD at vertical inputs to layer II/III pyramidal cells in rat barrel cortex. *Neuron* **27**: 45-56 <https://doi.org/10.1016/S0896-6273(00)00008-8>.

Froemke RC, Poo M-m and Dan Y (2005). Spike-timing-dependent synaptic plasticity depends on dendritic location. *Nature* **434**: 221-225 <https://doi.org/10.1038/nature03366>.

Joris PX, Smith PH and Yin TCT (1994). Enhancement of neural synchronization in the anteroventral cochlear nucleus. II. Responses in the tuning curve tail. *Journal of Neurophysiology* **71**: 1037-1051 <http://doi.org/10.1152/jn.1994.71.3.1037>.

Kuenzel T, Borst JGG and van der Heijden M (2011). Factors controlling the input-output relationship of spherical bushy cells in the gerbil cochlear nucleus. *J Neurosci* **31**: 4260-4273 <https://doi.org/10.1523/JNEUROSCI.5433-10.2011>.

Palmer AR and Shackleton TM (2009). Variation in the phase of response to low-frequency pure tones in the guinea pig auditory nerve as functions of stimulus level and frequency. *J Assoc Res Otolaryngol* **10**: 233-250 <https://doi.org/10.1007/s10162-008-0151-x>.

Sjöström PJ, Turrigiano GG and Nelson SB (2001). Rate, timing, and cooperativity jointly determine cortical synaptic plasticity. *Neuron* **32**: 1149-1164 <http://www.ncbi.nlm.nih.gov/pubmed/11754844>.

Spirou GA, Brownell WE and Zidanic M (1990). Recordings from cat trapezoid body and HRP labeling of globular bushy cell axons. *Journal of Neurophysiology* **63**: 1169-1190 <http://jn.physiology.org/content/63/5/1169.short>.

Versteegh CPC, Meenderink SWF and van der Heijden M (2011). Response characteristics in the apex of the gerbil cochlea studied through auditory nerve recordings. *J Assoc Res Otolaryngol* **12**: 301-316 <https://doi.org/10.1007/s10162-010-0255-y>.

Wei L, Karino S, Verschooten E and Joris PX (2017). Enhancement of phase-locking in rodents. I. An axonal recording study in gerbil. *J Neurophysiol* **118**: 2009-2023 <https://doi.org/10.1152/jn.00194.2016>.
